# Association of Healthcare and Aesthetic Procedures with Infections Caused by Nontuberculous Mycobacteria, France, 2012‒2020

**DOI:** 10.3201/eid2803.211791

**Published:** 2022-03

**Authors:** Côme Daniau, Emmanuel Lecorche, Faiza Mougari, Hanaa Benmansour, Claude Bernet, Hervé Blanchard, Jérôme Robert, Anne Berger-Carbonne, Emmanuelle Cambau

**Affiliations:** Santé Publique France, Saint-Maurice, France (C. Daniau, A. Berger-Carbonne);; Université de Paris, Paris, France (E. Lecorche, E. Cambau);; Assistance Publique Hôpitaux de Paris, Paris (E. Lecorche, F. Mougari, H. Benmansour, H. Blanchard, J. Robert, E. Cambau);; Centre National de Référence des Mycobactéries et de la Résistance des Mycobactéries aux Antituberculeux, Paris (E. Lecorche, F. Mougari, H. Benmansour, J. Robert, E. Cambau);; Centre d’Appui pour la Prévention des Infections Associées aux Soins en Provence-Alpes-Côte-d’Azur, Lyon, France (C. Bernet);; Centre d’Appui pour la Prévention des Infections Associées aux Soins en Île-de-France, Paris (H. Blanchard);; Centre d’Immunologie et des Maladies Infectieuses (CIMI),; Sorbonne Université, Paris (J. Robert)

**Keywords:** mycobacteria, bacteria, nontuberculous mycobacteria infections, tuberculosis and other mycobacteria, respiratory infections, healthcare-associated infections, aesthetic procedure‒associated infections, comparative genomics, epidemiology, case reports, retrospective study, France

## Abstract

We describe nontuberculous mycobacteria (NTM) infections during 2012–2020 associated with health care and aesthetic procedures in France. We obtained epidemiologic data from the national early warning response system for healtcare-associated infections and data on NTM isolates from the National Reference Center for Mycobacteria. We compared clinical and environmental isolates by using whole-genome sequencing. The 85 original cases were reported after surgery (48, 56%), other invasive procedures (28, 33%) and other procedures (9, 11%). NTM isolates belonged to rapidly growing (73, 86%) and slowly growing (10, 12%) species; in 2 cases, the species was not identified. We performed environmental investigations for 38 (45%) cases; results for 12 (32%) were positive for the same NTM species as for the infection. In 10 cases that had environmental and clinical samples whose genomes were similar, the infection source was probably the water used in the procedures. NTM infections could be preventable by using sterile water in all invasive procedures.

Nontuberculous mycobacteria (NTM) are ubiquitous bacteria found in soil, water, and other environments (*1*,*2*). More than 200 NTM species have been described to date. NTM are classified according to their speed of growth in vitro, specifically rapidly growing mycobacteria (such as *Mycobacterium chelonae*, *M. fortuitum* complex, and *M. abscessus*) and slowly growing mycobacteria (such as *M. avium* complex, *M. marinum*, and *M. kansasii*).

NTM infections are usually not transmissible between humans, although outbreaks linked to the same contamination event or from a common water reservoir have been reported. This finding was especially observed for extrapulmonary NTM infections after invasive procedures because of a common source, such as healthcare-associated infections (HAIs) and those related to medical, aesthetic, or cosmetic procedures (*3**–**7*). In particular, NTM HAIs were observed after heart surgery: >100 cases of endocarditis caused by a single clone of *M. chimaera* were found in water tanks of heater-cooler units used for cardiac bypass (*8*). In France, previously reported outbreaks of NTM HAI cases have involved *M. xenopi* in bone and joint infections after orthopedic surgery (*9*) and *M. chelonae* in skin infections after mesotherapy cosmetic procedures (*5*) or in hematopoietic stem cell transplantation (*10*). Endocarditis on bioprosthetic heart valves were also reported to contain *M. wolinskyi* and *M. chelonae* (*11*).

HAI reporting has been mandatory in France since 2001, and reports are collected at the French Public Health Agency. In addition, the National Reference Centre for Mycobacteria and Resistance of Mycobacteria to Anti-Tuberculosis Agents (CNR-MyRMA) regularly receives NTM isolates, including NTM HAI isolates, from human infections for diagnosis and treatment purposes. A previous case series described an initial cross-database evaluation focusing on NTM infections associated with cosmetic procedures during 2001–2010 (*12*). We describe episodes of extrapulmonary NTM infections associated with surgical, medical, or aesthetic procedures, including cosmetic care, reported in France during 2012‒2020, in and outside healthcare facilities (HCFs).

## Materials and Methods

### Data Sources

We used 2 data sources to include reported cases of NTM infections associated with surgical, medical, or aesthetic procedures during January 2012‒June 2020. The first data source was the national early warning response system (EWRS) for HAI diagnosed in HCF, using an electronic reporting process implemented in 2012 (e-SIN), and slightly modified in 2017 (*13*). The second data source was the NTM isolate database of the CNR-MyRMA, which includes microbiological results of clinical isolates, as well as environmental isolates found after epidemiologic investigations. The Regional Support Centre for the Prevention of Healthcare-Associated Infections conducted epidemiologic investigations. We contacted health professionals who reported cases to the national EWRS for HAI and send isolates to CNR-MyRMA to associate isolates with cases. We used this procedure to set up a single database containing epidemiologic and microbiological data.

### Case Definition

We included extrapulmonary NTM infections defined as a person who had clinical symptoms compatible with an NTM infection and >1 NTM-positive microbiological sample (cases considered as NTM colonization by physicians were excluded); and specific surgical, medical, or aesthetic procedures, including cosmetic care, potentially at the origin of the NTM infection. Pulmonary NTM infections, even hospital-acquired, were excluded. We usually consolidated epidemiologically related cases into a single report.

### Data Collection and Analysis

We collected the following information from the 2 data sources: 1) the report itself (the HCF or the laboratory which made the report, date of report); 2) data for infection (date of onset of initial symptoms, symptoms, infection type); 3) the context and suspected cause of the infection (procedure at the origin of the infection, date of contamination or invasive procedure, equipment implicated); 4) epidemic context (number of cases, distribution over time); 5) characteristics of case-patients (age, sex, and immune status); 6) investigation characteristics performed after the NTM infection diagnosis (environmental and professional practices investigations, corrective measures implemented); and 7) microbiological results (name of species and subspecies, whole-genome sequencing [WGS] comparison). We performed a descriptive data analysis by using STATA version 14.2 (https://www.stata.com). We analyzed the rate of NTM infection cases over the study period by using a Poisson regression model.

### Genomic Comparison

We performed genotypic analysis by using WGS to compare isolates found in environmental and clinical samples. We extracted DNA by using the DNA Ultraclean Microbial Kit (QIAGEN, https://www.qiagen.com). We prepared DNA libraries by using the Nextera XT Kit (Illumina, https://www.illumina.com) and sequenced them by using the MiSeq System (Illumina) and MiSeq Reagent V2 (2 × 150) Kits (Illumina). We performed WGS comparison by aligning sequencing reads of the isolates to a reference genome. We analyzed sequencing data by using Bionumerics version 7.6 (Applied Maths, https://www.applied-maths.com). We trimmed reads to exclude base calls with a Phred score <15 and then aligned them by using the Trimming and Resequencing analysis options. The single-nucleotide polymorphism (SNP) signature was built by using the Strict filtering (closed SNP set) option, retaining all SNP with a minimum coverage of 5×, at least covered once in both forward and reverse direction and a minimum distance between retained SNP position of 12 bases, removing the nondiscriminatory position. We used the SNP matrix to build a maximum parsimony tree. We defined a cluster in the WGS analysis by isolates sharing <10 or fewer SNPs. WGS data are available from the National Center for Biotechnology Information (https://dataview.ncbi.nlm.nih.gov; under BioProject nos. PRJNA597875, PRJNA657124, PRJNA576780, and PRJNA574109).

## Results

**Figure 1:**
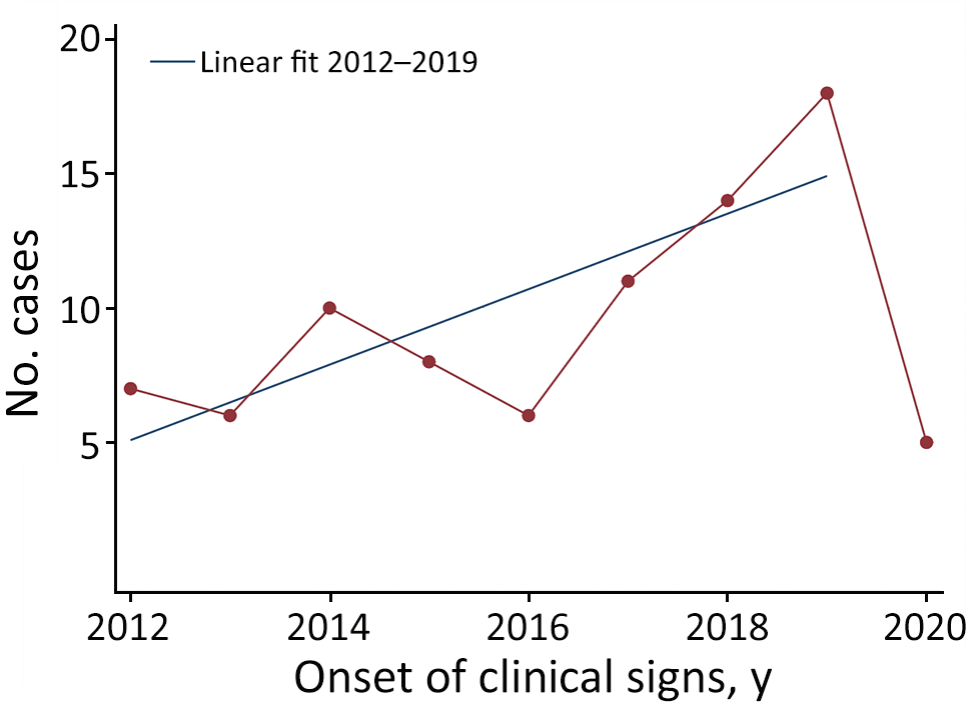
Onset of clinical signs in 85 reported cases of infection with nontuberculous mycobacteria associated with healthcare and aesthetic procedures, by year, France, January 2012‒June 2020. Blue line indicates linear fit for 2012‒2019 after excluding incomplete year 2020.

For the study period, 71 reports of extrapulmonary NTM infections related to HAI surgical, medical, or aesthetic procedures were included to give a total of 85 original cases, a mean of 10 cases/year for complete years (i.e., 2012–2019) (Figure 1). The regression identified an increasing trend of cases per year of onset of clinical signs during the study period after excluding incomplete years (p<0.01). This increase was observed particularly during the period 2016–2019 (i.e., after the *M. chimaera* heater–cooler unit [HCU] outbreak) but was caused by addition of these cases because regression analysis without them showed a similar significant trend (p<0.01). Among the 85 cases, 36 (42%) were found in the CNR-MyRMA strain database and the e-SIN database. The CNR-MyRMA database contained 30 additional cases, and the e-SIN database contained 19 additional cases.

The reports were received from 46 HCFs throughout France. Twenty-nine of the HCFs sent only 1 report during the study period, and 16 HCFs sent >1 (10 HCFs made 2 reports, 3 HCFs made 3 reports, 2 HCFs made 4 reports, and 1 HCF made 5 reports). Most (90%, 64/71) of the reports concerned an individual case, and 7 reports concerned clusters (2 cases in 4 reports, 3 cases in 1 report, and 5 cases in 2 reports).

For most (69%, 59/85) of cases, the infection was acquired inside the HCF, whereas 26 cases (31%) were acquired outside the reporting HCF. These infections acquired outside the HCF were imported either from another HCF (n = 14) or from a non–hospital-based medical or aesthetic practice (n = 12).

More women than men had cases reported (M:F = 31:46); sex was not reported for 8 case-patients. The median case age was 54 years (range 4–86 years; age was not reported for 12 cases). One third of the cases concerned immunosuppressed patients (33%, 23/70 cases; 15 cases did not report this information). NTM infections resulted from surgical procedures (56%, 48/85 cases), other invasive procedures (33%, 28/85 cases), and noninvasive procedures (11%, 9/85 cases). Cardiovascular surgery (n = 14), orthopedic surgery (n = 11), plastic surgery (e.g., breast surgery, (n = 13), face-lift (n = 2), abdominoplasty (n = 1), or capillary implant (n = 1), and catheter-associated infections (n = 17) comprised most of the surgical and other invasive procedure cases reported. The NTM infections concerned mainly skin and soft tissues (36%, 31/85), intravascular catheters (20%, 17/85), bones and joints (18%, 15/85), and arterial/cardiac (15%, 13/85) (Table 1).

**Table 1:** Description of 85 reported healthcare and aesthetic-associated NTM infections, France, 2012–2020*

Procedure	Infection type	Sex, M/F	Median age, y (range)	Infection risk factor	Median incubation times, d (range)	Species implicated
Surgical, n = 48						
Cardiovascular surgery, n = 14	Infective endocarditis and aortic infection, n = 13	7/3, NR = 3	69 (47‒81); n = 10; NR, n = 3	None, n = 10; NR, n = 3	393 (14‒732); n = 11; NR, n = 2	*M. chelonae*, n = 5; *M. chimaera*, n = 3; AFB positive and culture negative, n = 2; *M. wolinskyi*, n = 1; *M. avium*, n = 1; *M. abscessus*,† n = 1; *M. lentiflavum*,† n = 1; *M. fuerthensis*,† n = 1
	SST, n = 1	0/1	69	None	41	*M. fortuitum complex*, n = 1
Breast surgery, n = 13	SST	1/12	42 (31‒53); n = 10; NR, n = 3	Breast cancer, n = 2; HIV, n = 1; none, n = 8; NR, n = 2	36 (10‒732); n = 12; NR, n = 1	*M. fortuitum*, n = 6; *M. abscessus*, n = 3; *M. senegalense*, n = 1; *M. chelonae*, n = 1; *M. avium*, n = 1; *M. chimaera*, n = 1
Orthopedic Surgery, n = 11	Bone and joint	5/2, NR = 4	69 (45‒86); n = 7; NR, n = 4	None, n = 6; NR, n = 5	33 (23‒183); n = 9, NR, n = 2	*M. fortuitum*, n = 4; *M. abscessus*, n = 3; *M. mageritense*, n = 2; *M. porcinum*, n = 1; *M. chelonae*, n = 1
Skin surgery,‡ n = 7	SST	4/3	65 (42‒78); n = 6; NR, n = 1	None, n = 6; NR, n = 1	45 (30‒93); n = 6, NR, n = 1	*M. fortuitum*, n = 3; *M. chelonae*, n = 2; *M. abscessus*, n = 1; *M. neoaurum*, n = 1
Other surgery,§ n = 3	Vascular, n = 1	NR	NR	NR	NR	*M. fortuitum*, n = 1
	Urogenital, n = 1	0/1	48	None	NR	*M. fortuitum*, n = 1
	Ocular, n = 1	0/1	83	None	15	*M. chelonae*, n = 1
Invasive, n = 28						
Vascular catheter insertion, n = 17	Intravascular catheter	7/10	58 (4‒82); n = 17	Chemotherapy, n = 14; none, n = 2; NR = 1	NR	*M. chelonae*, n = 6; *M. mucogenicum*, n = 5; *M. abscessus*, n = 2; *M. fortuitum*, n = 1; *M. fortuitum complex*, n = 1; *M. porcinum*, n = 1; *M. neoaurum*, n = 1
Infiltration, n = 3	Bone and joint	0/3	52 (52‒84); n = 3	Corticosteroid infiltration, n = 3	61 (33‒108); n = 3	*M. abscessus*, n = 2; *M. chelonae*, n = 1
Mesotherapy, n = 3	SST	1/2	47 (35‒49); n = 3	None, n = 3	30 (15‒73); n = 3	*M. chelonae*, n = 2; *M. abscessus*, n = 1
Tattoo, n = 3	SST	3/0	50 (48‒56); n = 3	None, n = 3	NR	*M. chelonae*, n = 3
Intestinal endoscopy, n = 2	Abdominal	0/2	68 (60‒75); n = 2	Kidney transplant, n = 1; none, n = 1	7 (4‒10); n = 2	*M. fortuitum*, n = 1; *M. abscessus*, n = 1
Noninvasive, n = 4						
Eye lens use, n = 3	Ocular	0/3	36 (21‒62); n = 3	None, n = 3	NR	*M. chelonae*, n = 3
Balneotherapy, n = 1	SST	0/1	51	Methotrexate plus corticosteroids treatment	20	*M. marinum*, n = 1
Not identified, n = 5						
	SST, n = 3	1/2	44 (28‒75); n = 3	Kidney transplant, n = 1; NR = 2	NR	*M. chelonae*, n = 3
	Disseminated, n = 1	1/0	64	Corticosteroids treatment, chronic dialysis	NR	*M. chelonae*, n = 1
	Bone and joint, n = 1	1/0	61	Corticosteroids treatment	NR	*M. chelonae*, n = 1

*AFB, acid-fast bacilli; NR, not reported; NTM, nontuberculous mycobacteria; SST, akin and soft tissue. †Sample that had several bacteria identified. ‡Skin operations concerning the following diverse procedures: face-lift, n = 2, abdominoplasty, n = 1, capillary implant, n = 1, excision of a basal cell carcinoma, n = 1, wearing a Holter monitor, n = 1 and neurostimulation device, n = 1. §Lower limb vascular surgery, n = 1, promontofixation, n = 1, eye surgery, n = 1.

Overall, 14 NTM species were isolated: 10 rapidly growing NTM species and 4 slowly growing NTM species. Rapidly growing NTM were *M. chelonae* (n = 30), *M. fortuitum complex* (n = 24) (17 cases with *M. fortuitum*, 2 with *M. mageritense*, 2 with *M. porcinum*, 1 with *M. senegalense*, and 2 strains for which the exact species could not be determined), *M. abscessus* (n = 14), *M. mucogenicum* (n = 5), *M. neoaurum* (n = 2), *M. fuerthensis* (n = 1), and *M. wolinskyi* (n = 1). Slowly growing NTM species were *M. chimaera* (n = 4), *M. avium* (n = 2), *M. lentiflavum* (n = 1), and *M. marinum* (n = 1). For 2 cases of endocarditis, direct examination of the valve samples identified acid-fast bacilli after Ziehl-Neelsen staining, but culture results were negative.

We determined the hypothesized incubation time (i.e., time between the onset of clinical signs and most probable contamination date, which is most often the date of the procedure) for 50 cases (Table 1). Incubation time was shorter for rapidly growing NTM (median time 34 days, n = 40) than for slowly growing NTM (median time 549 days, n = 5).

We suspected that medical devices were related to the infection for 80% of cases (68/85), but a medical device vigilance report was performed for only 21% of those (14/68 cases). Medical devices comprised implantable devices (e.g., breast prosthesis, artificial heart valve and vascular prosthesis, knee or hip prosthesis) for 50% of case-patients that had a medical device (34/68 cases), invasive devices (e.g., catheter and implantable port, dialysis device, endoscopy device, infiltration device in orthopedic surgery, liposuction cannula, mesotherapy, and tattoo injection equipment) (37%, 25/68 cases), and noninvasive devices (e.g., cardiopulmonary bypass HCU, contact lens) (13%, 9/68 cases).

For nearly half of all reported cases (47%, 40/85 cases; 30 cases did not report this information), there was a specific investigation of professional practices after the NTM infection was diagnosed to assess the level of compliance with hygiene guidelines, sterilization procedures, and treatment procedures. For more than one fourth of these cases (28%, 11/40), the investigations found failure to comply with infection risk prevention recommendations. Corrective measures were implemented for 45% of the reports (17/38 reports; 33 did not report this information), most often involving increased hygiene vigilance and recommendations to improve practices (n = 7). For 32 reports, there was no active case finding; most of them were isolated cases. For 30 reports, this information was not available. Active case finding was conducted after 9 reports (30 did not report this information) for procedures such as breast reconstruction, heart surgery, gastrointestinal endoscopy, mesotherapy, orthopedic surgery, and tattooing sessions.

**Figure 2:**
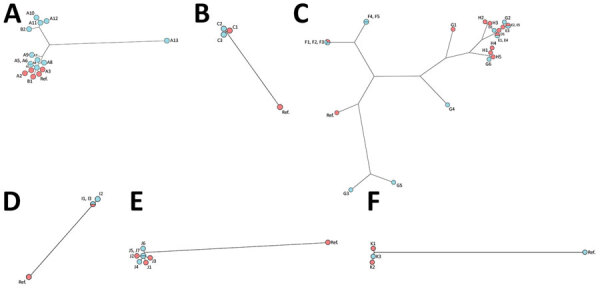
Genomic comparison of nontuberculous mycobacteria isolates by using whole-genome sequencing phylogenetic analysis and maximum parsimony trees. A) *Mycobacterium chimaera*, B) *M. fortuitum*, C) *M. chelonae*, D) *M. marinum*, E) *M. mucogenicum*, F) *M. neoaurum*. Environmental isolates are indicated in blue, and clinical isolates are indicated in red. Additional information for the 6 *Mycobacterium* species tested is provided in the Appendix. Ref, referent. Panel A: Based on 19,621 single-nucleotide polymorphisms (SNPs) generated from comparison of 16 genomes using square root scaling. Isolates were 1) clinical isolates from 2 patients who had *M. chimaera* disseminated disease after open-heart surgery (A1 and A2 from the first patient), A3 from the second patient; 2) a reference genome from a previously described epidemic patient (*8*); 3) 10 heater-cooler unit water samples from the hospital where the second patient underwent surgery, collected 4 years after the report (A4‒A13); 4) clinical sample from a patient who had a breast prosthesis infection (B1); and 5) the environmental isolate collected in the hospital’s water network 1 year after the report of the breast prosthesis infection (B2). Panel B: Based on 27,796 SNPs generated from comparison of 4 genomes using square root scaling. Isolates were 1) clinical isolate from a patient who had *M. fortuitum* breast prosthesis infection (C1) and 2) environmental isolate collected 1 month after the report in the water supply network in the shower of the patient’s hospital room (C2, C3). Panel C: Based on 67,759 SNPs generated from comparison of 24 genomes using square root scaling. Isolates were 11 clinical isolates: 1 from an infection after face lift surgery (D1), 3 from 2 skin and soft tissue infections after a tattoo (E1‒E2 from the same patient and E3 from another patient who was tattooed in the same tattoo parlor); 2 from 2 mesotherapy infections from 2 nonrelated reports (F1 and G1); and 5 using supply networks were obtained for investigations including: 1 isolate from a surgical sink (D2), 4 isolates from a doctor’s office sink (F2‒F3, G5‒G6), 2 isolates from a tattoo parlor’s sink used to dilute the ink (E4‒E5) and 5 isolates from a patient’s home (F4‒F5, G2‒G4). Panel D: Based on 24,757 SNPs generated from comparison of 4 genomes using square root scaling. Isolates were 1) a clinical isolate from a skin and soft tissue infection caused by contamination after a bath in a balneotherapy swimming pool (I1 and I2) 2 environmental isolates from the swimming pool (I2 and I3). Panel E: Based on 53,551 SNPs generated by comparison of 8 genomes using square root scaling. Isolates were 1) 3 clinical isolates from catheter-associated infections (3 patients J1‒J3) and 2) 4 environmental isolates from hospital water supply networks (J4‒J7). Panel F: Based on 58,473 SNPs generated by comparison of 4 genomes using square root scaling. Isolates were 1) 2 clinical isolates from the blood culture (K1 and K2) of 1 patient and 2) 1 isolate from microbiological control after an autologous stem cell transplant (K3).

Environmental investigations were undertaken for 42% (30/71) of the reports. Most (27/30) reports involved water sampling from potential sources of the contamination, such as water supply networks (n = 21), HCU (n = 4), dialysis water (n = 1), and swimming pool water (n = 1). Perioperative surfaces (n = 2) and air samples (n = 1) were rarely sampled. Among the 45% of cases (38/85) involving environmental investigations, mycobacteria samples were positive for 18 (7 were not reported). The same NTM species as in the clinical isolate was found for 12 cases (32%, 12/38) after environmental investigations. For 10 cases, the clinical isolate could not be distinguished from the environmental isolate (Table 2; Figure 2): *M. chimaera* isolates from HCU and heart surgery infection (patient A3) (Figure 2, panel A), isolates from hospital water supply network, *M. fortuitum* breast infection (patient C1) (Figure 2, panel B), *M. chelonae* skin and soft tissue infection (patients D1, E1-E2, E3, and F1) (Figure 2, panel C), *M. marinum* isolates from pool balneotherapy and skin and soft tissue infection (patient I1) (Figure 2, panel D), and *M. mucogenicum* catheter-associated infection (patients J1, J2, and J3) (Figure 2, panel E).

**Table 2:** Genomic comparison between clinical versus environmental isolates and comparison of clinical isolates for patients suspected of being contaminated with nontuberculous mycobacteria by a common source, France, 2012–2020

Report	Species involved	Case manifestations	Environmental sample	Result of comparison	Location of information*
A	*M. chimaera*	Endocarditis after cardiac surgery by using contaminated heater-cooler unit (2 patients operated on in 2 hospitals)	Heater-cooler unit water	Clinical isolates from the 2 patients who had *M. chimaera* disseminated disease after open-heart surgery belonged to worldwide epidemic cluster. Environmental isolates, obtained only for 1 of the 2 patients, belonged to the epidemic cluster for 5/10 of them	Figure 2, panel A; Appendix Table 1
B	*M. chimaera*	Prosthesis infection after breast reconstruction (1 patient)	Hospital water supply network	Environmental and clinical isolates did not belong to the same cluster	Figure 2, panel A; Appendix Table 1
C	*M. fortuitum*	Prosthesis infection after breast reconstruction (1 patient)	Hospital water supply network	Environmental and clinical isolates belonged to the same cluster	Figure 2, panel B; Appendix Table 2
D	*M. chelonae*	Skin and soft tissue infection after face lift surgery (1 patient)	Hospital water supply network	Environmental and clinical isolates belonged to the same cluster	Figure 2, panel C; Appendix Table 3
E	*M. chelonae*	Skin and soft tissue infection after tattoo (2 patients tattooed in the same tattoo parlor)	Tattoo parlor water supply network	Environmental and clinical isolates belonged to the same cluster	Figure 2, panel C; Appendix Table 3
F	*M. chelonae*	Skin and soft tissue infection after mesotherapy (1 patient)	Water supply network from doctor’s office sink and patient’s home	Environmental isolates from doctor’s office sink and clinical isolate belonged to the same cluster. Isolates from patient’s home were not related	Figure 2, panel C; Appendix Table 3
G	*M. chelonae*	Skin and soft tissue infection after mesotherapy (1 patient)	Water supply network from doctor’s office sink and patient’s home	Environmental and clinical isolates did not belong to the same cluster	Figure 2, panel C; Appendix Table 3
H	*M. chelonae*	Catheter-associated infection (5 patients from the same institution)	No environmental sample	Two clusters of 2 clinical isolates were identified	Figure 2, panel C; Appendix Table 3
I	*M. marinum*	Skin and soft tissue infection caused by contamination after a bath in a balneotherapy swimming pool (1 patient)	Swimming pool water	Environmental and clinical isolates belong to the same cluster	Figure 2, panel D; Appendix Table 4
J	*M. mucogenicum*	Catheter-associated infection (3 patients from the same institution)	Hospital water supply network	Environmental and clinical isolates belong to the same cluster	Figure 2, panel D; Appendix Table 5
K	*M. neoaurum*	Catheter-associated infection discovered during microbiological control of autologous stem cell transplant (1 patient)	Autologous stem cell transplant; no environmental sample	Environmental and clinical isolates belong to the same cluster	Figure 2, panel E; Appendix Table 6
*Appendix.					

We also performed genomic comparisons for case-patients suspected of being contaminated by a common source. For 8/10 case-patients, studied isolates had the same pattern (Table 2; Figure 2). In report A, 3 clinical isolates of *M. chimaera* endocarditis from 2 patients were clustered (A1-A2 and A3) (Figure 2, panel A). These 2 patients were linked to a worldwide outbreak of HCU contamination, as shown in the section comparing clinical (A3) and environmental (A4-A8) isolates. In report H, which concerned *M. chelonae* catheter-associated infections diagnosed in the same institution (n = 5 cases), clinical isolates were clustered into 2 distinct groups; the first cluster grouped 3 isolates, H1, H4, and H5, from 3 patients, and the second cluster grouped 2 isolates, H2 and H3, from 2 patients (Figure 2, panel C). The presence of 2 clusters in the same HCF suggested that there were 2 sources of contamination, neither of which were found. In report K, the same genotype was found when comparing *M. neoaurum* isolates from blood cultures of 1 patient with the isolate found during microbiological control testing after a peripheral autologous stem cell transplant (n = 1 case) (Figure 2, panel F). The contamination of the stem cell transplant was attributed to colonization of the catheter used for the cell sampling.

## Discussion

We describe extrapulmonary NTM infections diagnosed after surgical, medical, or aesthetic procedures in France over an 8-year period (2012–2020). To broaden the spontaneous reporting from medical professionals, we sought 2 information sources: the national EWRS for HAI and the national reference NTM strain database. Because only 85 cases were described in 71 reports over 8 years, we might consider that such NTM infections remain rare. However, most of the cases were related to a medical device, a specific procedure, or lack of hygiene practices and might have been preventable.

Our study highlights a slight increase in reported annual numbers of cases during the study period. This increase, particularly during 2016 (*14*), could be explained by greater global awareness in public health community after invasive infections with *M. chimaera* associated with HCUs used during cardiac surgery (*15*) and other published outbreaks (*6*).

Both infection sites and NTM species isolated from these cases of infections were diverse, emphasizing the opportunistic nature of these pathogens. The most commonly reported infections were skin and soft tissue infections, catheter-related infections, infective endocarditis, and bone and joint infections. When we compared our findings with the major proportion of extrapulmonary NTM infections described in the literature, but not limited to healthcare-associated and aesthetic procedure‒associated infections, we found that skin and soft tissue infections were the most commonly reported infection site (*16*,*17*). However, we identified catheter-associated infections, bone and joint infections, or infective endocarditis, which are less described, except for the HCU *M.* *chimaera* outbreak (*8*).

A wide variety of NTM species were responsible for the infections reported in our study; *M. chelonae* and *M. fortuitum* were the most common. Isolates of slowly growing NTM species were rare (11%) compared with those found in a review in the United States, in which 50% of all species were in the *M. avium* complex (*16*). One possible explanation for this difference is that rapidly growing NTM cultivated on standard bacteriology diagnostic media might constitute an unexpected etiology diagnosis. We should also consider that these rapidly growing species were regularly found in France in the water networks, one of the sources of infection (*1*).

Most of the cases in our study were linked to surgical, invasive, and noninvasive procedures. In 5 cases, no specific procedures were identified as the cause of infection, even though all patients underwent healthcare procedures such as previous intravenous catheter, or no procedures were identified at all, such as the case of bone and joint infection, which could not have originated from spontaneous infection. As observed in previous reviews, the most commonly reported infections were those associated with aesthetic care (*12*), particularly breast prostheses (*18*).

The source of infection was not determined in 9/10 case-patients, despite environmental investigations conducted for ≈50% of the reports. Even when environmental investigations were performed, they occurred months after the suspected contamination because of long incubation times, as in reports B and G (Table 2).

Because a medical device was implicated in most of the NTM infection cases we reported, we believe that these infections can be prevented. The medical device could be contaminated by NTM before its use or can lead to contamination from environmental NTM (*5*,*19*). When an environmental cause is identified, the water system is the major environmental source most frequently considered responsible. Water systems, particularly in hospitals, are frequently identified as NTM reservoirs (*20*,*21*).

Genomic comparison of NTM isolates can be used to rule out or confirm any hypothesis concerning the origin of the contamination. However, careful analysis of genomic sequence comparisons should be conducted because several factors, such as the reference sequence on which the reads are mapped (epidemic strain or unrelated strain), quality of the sequenced data, coverage of the mapping assembly, number of sequences included in the comparison, and use of de novo assembly, influence SNP analysis. WGS appears to be a suitable tool for the molecular investigation of NTM infections, but might need expert rules and standardization to be used further.

The major limitations of this study concern the lack of completeness of the reported data. There is no specific surveillance system for NTM infection in France, and the 2 databases used for this case series are not exhaustive. The purpose of the EWRS for HAI platform is to improve the management of HAI reporting by HCF, and the CNR-MyRMA receives NTM isolates for patient diagnosis and treatment and genotypic comparison in epidemiologically related cases with environmental analyses when necessary. Therefore, underdeclaration of NTM infection cases in France is probable (*22*). However, when combined, the 2 databases provide a useful inventory of extrapulmonary NTM infection cases related to surgical, medical, and aesthetic procedures. The genomic comparison of NTM isolates performed by CNR-MyRMA was able to demonstrate the source when an environmental investigation was conducted and clinical and environmental isolates were available. This comparison provides valuable pointers for the future implementation, improvement, and follow-up of certain preventive measures.

Although data in the current study were not exhaustive, reports of NTM infection cases and subsequent microbiological and workplace practice investigations showed that considerable progress has been made in understanding contamination mechanisms during healthcare treatment. Water used in the procedures appeared to be the infection source for 10 cases. This finding is particularly true for heart surgery after the alert issued concerning the global outbreak of *M. chimaera* endocarditis as a result of contaminated HCUs (*8*).

Our observations should prompt more stringent recommendations for prompt reporting of NTM infections and provision of clinical and environmental samples for analysis of strains. Better application of these recommendations should improve methods to identify causes of NTM infections and enable their prevention.

Appendix Additional information on association of healthcare and aesthetic procedures with infections caused by nontuberculous mycobacteria, France, 2021‒2020.

## References

[1] Le Dantec C, Duguet JP, Montiel A, Dumoutier N, Dubrou S, Vincent V. Occurrence of mycobacteria in water treatment lines and in water distribution systems. Appl Environ Microbiol. 2002;68:5318–25. 10.1128/AEM.68.11.5318-5325.2002 12406720 PMC129932

[2] Falkinham JO III. Environmental sources of nontuberculous mycobacteria. Clin Chest Med. 2015;36:35–41. 10.1016/j.ccm.2014.10.003 25676517

[3] Meyers H, Brown-Elliott BA, Moore D, Curry J, Truong C, Zhang Y, et al. An outbreak of *Mycobacterium chelonae* infection following liposuction. Clin Infect Dis. 2002;34:1500–7. 10.1086/340399 12015697

[4] Conaglen PD, Laurenson IF, Sergeant A, Thorn SN, Rayner A, Stevenson J. Systematic review of tattoo-associated skin infection with rapidly growing mycobacteria and public health investigation of a cluster in Scotland, 2010. Euro Surveill. 2013;18:20553. 10.2807/1560-7917.ES2013.18.32.20553 23968828

[5] Carbonne A, Brossier F, Arnaud I, Bougmiza I, Caumes E, Meningaud JP, et al. Outbreak of nontuberculous mycobacterial subcutaneous infections related to multiple mesotherapy injections. J Clin Microbiol. 2009;47:1961–4. 10.1128/JCM.00196-09 19386853 PMC2691096

[6] Leão SC, Viana-Niero C, Matsumoto CK, Lima KV, Lopes ML, Palaci M, et al. Epidemic of surgical-site infections by a single clone of rapidly growing mycobacteria in Brazil. Future Microbiol. 2010;5:971–80. 10.2217/fmb.10.49 20521940

[7] Mora AD, Giraldo S, Castillo DA, Ferro BE. [Clinical behavior of infection and disease caused by non-tuberculous mycobacteria in Latin America: Scoping review] [in Spanish]. Rev Peru Med Exp Salud Publica. 2021;38:318–25. 10.17843/rpmesp.2021.382.6108 34468583

[8] van Ingen J, Kohl TA, Kranzer K, Hasse B, Keller PM, Katarzyna Szafrańska A, et al. Global outbreak of severe *Mycobacterium chimaera* disease after cardiac surgery: a molecular epidemiological study. Lancet Infect Dis. 2017;17:1033–41. 10.1016/S1473-3099(17)30324-9 28711585

[9] Astagneau P, Desplaces N, Vincent V, Chicheportiche V, Botherel A, Maugat S, et al. *Mycobacterium xenopi* spinal infections after discovertebral surgery: investigation and screening of a large outbreak. Lancet. 2001;358:747–51. 10.1016/S0140-6736(01)05843-3 11551599

[10] Ferry C, Saussine A, Bouaziz JD, Xhaard A, Peffault de Latour R, Ribaud P, et al. Disseminated cutaneous infection due to *Mycobacterium chelonae* following hematopoietic stem cell transplantation. IDCases. 2014;1:68–9. 10.1016/j.idcr.2014.07.002 26839776 PMC4735022

[11] Yuan SM. Mycobacterial endocarditis: a comprehensive review. Rev Bras Cir Cardiovasc. 2015;30:93–103. 25859873 10.5935/1678-9741.20140113 PMC4389517

[12] Couderc C, Carbonne A, Thiolet JM, Brossier F, Savey A, Bernet C, et al. [Non-tuberculous mycobacterial infections related to esthetic care in France, 2001-2010] [in French]. Med Mal Infect. 2011;41:379–83. 10.1016/j.medmal.2011.02.007 21440389

[13] Ministry of Social Affairs and Health. Decree no. 2017–129 of February 3, 2017 relating to the prevention of healthcare-associated infections, 2017 [in French] [cited 2021 Dec 9]. https://www.legifrance.gouv.fr/jorf/id/JORFTEXT000033982071

[14] Rajendran P, Padmapriyadarsini C, Mondal R. Nontuberculous mycobacterium: An emerging pathogen: Indian perspective. Int J Mycobacteriol. 2021;10:217–27. 34494559 10.4103/ijmy.ijmy_141_21

[15] European Center for Disease Prevention and Control. Rapid risk assessment: invasive cardiovascular infection by *Mycobacterium chimaera* potentially associated with heater-cooler units used during cardiac surgery, April 30, 2015 [cited 2021 Jun 28]. https://ecdc.europa.eu/en/publications-data/invasive-cardiovascular-infection-mycobacterium-chimaera-potentially-associated

[16] Henkle E, Hedberg K, Schafer SD, Winthrop KL. Surveillance of extrapulmonary nontuberculous mycobacteria infections, Oregon, USA, 2007‒2012. Emerg Infect Dis. 2017;23:1627–30. 10.3201/eid2310.170845 28930014 PMC5621539

[17] Blanc P, Dutronc H, Peuchant O, Dauchy FA, Cazanave C, Neau D, et al. Nontuberculous mycobacterial infections in a French hospital: a 12-year retrospective study. PLoS One. 2016;11:e0168290. 10.1371/journal.pone.0168290 27959960 PMC5154556

[18] Jaubert J, Mougari F, Picot S, Boukerrou M, Barau G, Ali Ahmed SA, et al. A case of postoperative breast infection by *Mycobacterium fortuitum* associated with the hospital water supply. Am J Infect Control. 2015;43:406–8. 10.1016/j.ajic.2014.12.023 25838135

[19] Regnier S, Cambau E, Meningaud JP, Guihot A, Deforges L, Carbonne A, et al. Clinical management of rapidly growing mycobacterial cutaneous infections in patients after mesotherapy. Clin Infect Dis. 2009;49:1358–64. 10.1086/606050 19814609

[20] Donohue MJ, Mistry JH, Donohue JM, O’Connell K, King D, Byran J, et al. Increased frequency of nontuberculous mycobacteria detection at potable water taps within the United States. Environ Sci Technol. 2015;49:6127–33. 10.1021/acs.est.5b00496 25902261

[21] Li T, Abebe LS, Cronk R, Bartram J. A systematic review of waterborne infections from nontuberculous mycobacteria in health care facility water systems. Int J Hyg Environ Health. 2017;220:611–20. 10.1016/j.ijheh.2016.12.002 28017547

[22] Regnier S, Caumes E. [Non-tuberculous mycobacterial infections related to esthetic care in France, 2001-2010] [in French]. Med Mal Infect. 2011;41:667–8. 10.1016/j.medmal.2011.09.009 22019499

