# Supplementary material for: Association of Healthcare and Aesthetic Procedures with Infections Caused by Nontuberculous Mycobacteria, France, 2012‒2020
Source: Emerg Infect Dis. 2022 Mar;28(3):518–26. doi: 10.3201/eid2803.211791 (PMC8888244; doi:10.3201/eid2803.211791)
Supplement: Appendix — Additional information on association of healthcare and aesthetic procedures with infections caused by nontuberculous mycobacteria, France, 2021‒2020. [file 21-1791-Techapp-s1.pdf]

Article DOI: https://doi.org/10.3201/eid2803.211791

Association of Healthcare and Aesthetic Procedures with Infections Caused by Nontuberculous Mycobacteria, France, 2012–2020

Appendix

Appendix Table 1. SNP matrix of *Mycobacterium chimaera* isolates*

Isolate	Mean coverage (X)	A3	A4	A1	A5	A6	Referent	A2	A7	A8	B1	A9	A10	A11	A12	B2	A13
A3	78	0															
A4	65	1	0														
A1	78	2	1	0													
A5	54	3	2	3	0												
A6	68	3	2	3	0	0											
Referent	–	4	3	4	5	5	0										
A2	48	6	5	4	7	7	8	0									
A7	58	7	6	7	8	8	9	11	0								
A8	60	10	9	10	11	11	12	14	7	0							
B1	64	30	29	30	31	31	32	34	35	38	0						
A9	75	166	165	166	167	167	168	170	161	156	194	0					
A10	57	929	928	929	930	930	931	933	924	923	957	901	0				
A11	59	927	926	927	928	928	929	931	922	921	955	899	2	0			
A12	53	929	928	929	930	930	931	933	924	923	957	901	4	2	0		
B2	28	1002	1001	1002	1003	1003	1004	1006	997	996	1030	968	87	85	87	0	
A13	63	18832	18831	18832	18833	18833	18834	18836	18832	18831	18860	18888	19130	19128	19130	19199	0

*Reads were mapped to the *M. chimaera* ZUERICH-1 strain genome (CP015272.1) (*1*). More information on A1-A2 isolates is available (*2*). SNP, single-nucleotide polymorphism.

Appendix Table 2. SNP matrix of *Mycobacterium fortuitum* isolates*

Isolate	Mean coverage (×)	C3	C2	C1	Referent
C3	66	0			
C2	84	3	0		
C1	41	5	4	0	
Referent	–	>20,000	>20,000	>20,000	0

*Reads were mapped to the *M. fortuitum* DSM 46,621 strain genome (*3*). SNP, single-nucleotide polymorphism.

Appendix Table 3. SNP matrix of *Mycobacterium chelonae* isolates*

Isolate	Mean coverage (×)	E4	E1	D2	E5	E2	E3	D1	G2	H2	H3	H1	H4	G6	H5	G1	G4	F4	F5	F2	F3	F1	Ref	G5	G3
E4	54	0																							
E1	62	0	0																						
D2	69	5	5	0																					
E5	55	8	8	11	0																				
E2	54	8	8	11	0	0																			
E3	54	7	7	10	1	1	0																		
D1	75	5	5	8	3	3	2	0																	
G2	58	9	9	12	3	3	2	4	0																
H2	54	2539	2539	2542	2545	2545	2544	2542	2546	0															
H3	60	2538	2538	2541	2544	2544	2543	2541	2545	3	0														
H1	54	4573	4573	4576	4579	4579	4578	4576	458	4622	4621	0													
H4	50	4574	4574	4577	4580	4580	4579	4577	4581	4623	4622	1	0												
G6	62	4574	4574	4577	4580	4580	4579	4577	4581	4623	4622	9	10	0											
H5	60	4576	4576	4579	4582	4582	4581	4579	4583	4625	4624	11	12	10	0										
G1	63	8073	8073	8076	8078	8078	8077	8075	8079	8102	8101	7925	7926	7926	7928	0									
G4	73	14935	14935	14938	1494	1494	14939	14937	14941	14951	1495	14864	14865	14865	14867	14127	0								
F4	32	>20000	>20000	>20000	>20000	>20000	>20000	>20000	>20000	>20000	>20000	>20000	>20000	>20000	>20000	>20000	>20000	0							
F5	32	>20000	>20000	>20000	>20000	>20000	>20000	>20000	>20000	>20000	>20000	>20000	>20000	>20000	>20000	>20000	>20000	0	0						
F2	29	>20000	>20000	>20000	>20000	>20000	>20000	>20000	>20000	>20000	>20000	>20000	>20000	>20000	>20000	>20000	>20000	4665	4665	0					
F3	32	>20000	>20000	>20000	>20000	>20000	>20000	>20000	>20000	>20000	>20000	>20000	>20000	>20000	>20000	>20000	>20000	4665	4665	0	0				
F1	30	>20000	>20000	>20000	>20000	>20000	>20000	>20000	>20000	>20000	>20000	>20000	>20000	>20000	>20000	>20000	>20000	4665	4665	0	0	0			
Ref	–	>20000	>20000	>20000	>20000	>20000	>20000	>20000	>20000	>20000	>20000	>20000	>20000	>20000	>20000	>20000	>20000	14535	14535	13241	13241	13241	0		
G5	32	>20000	>20000	>20000	>20000	>20000	>20000	>20000	>20000	>20000	>20000	>20000	>20000	>20000	>20000	>20000	>20000	>20000	>20000	>20000	>20000	>20000	>20000	0	
G3	34	>20000	>20000	>20000	>20000	>20000	>20000	>20000	>20000	>20000	>20000	>20000	>20000	>20000	>20000	>20000	>20000	>20000	>20000	>20000	>20000	>20000	>20000	4724	0

*Reads were mapped to the *M. chelonae* CCUG 47,445 strain genome (CP007220.1) (*4*). Ref, referent; SNP, single-nucleotide polymorphism.

Appendix Table 4. SNP matrix of *Mycobacterium marinum* isolates*

Isolate	Mean coverage (×)	I1	I3	I2	Referent
I1	97	0			
I3	83	0	0		
I2	87	2	2	0	
Referent	–	>20,000	>20,000	>20,000	0

*Reads were mapped to the *M. marinum* strain M genome (*5*). SNP, single-nucleotide polymorphism.

Appendix Table 5. SNP matrix of *Mycobacterium mucogenicum* isolates*

Isolate	Mean coverage (×)	J5	J7	J2	J6	J4	J3	J1	Referent
J5	24	0							
J7	27	0	0						
J2	26	1	1	0					
J6	25	2	2	3	0				
J4	30	3	3	4	5	0			
J3	37	3	3	4	5	6	0		
J1	23	4	4	5	6	7	7	0	
Referent	–	>20,000	>20,000	>20,000	>20,000	>20,000	>20,000	>20,000	0

*Reads were mapped to the *M. mucogenicum* strain DSM 44,124 genome (NZ_POTL01000001, BioProject: PRJNA224116, BioSample: SAMN08339096, Leif A. Kirsebom, Department of Cell and Molecular Biology, Uppsala University, Uppsala, Sweden. SNP, single-nucleotide polymorphism.

Appendix Table 6. SNP matrix of *Mycobacterium neoaurum* isolates*

Isolate	Mean coverage (×)	K3	K2	K1	Referent
K3	34	0			
K2	28	1	0		
K1	28	6	7	0	
Referent	–	>20,000	>20,000	>20,000	0

*Reads were mapped to the *M. neoaurum* strain HGMS2 genome (*6*). SNP, single-nucleotide polymorphism.

References

1. van Ingen J, Kohl TA, Kranzer K, Hasse B, Keller PM, Katarzyna Szafrańska A, et al. Global outbreak of severe *Mycobacterium* chimaera disease after cardiac surgery: a molecular epidemiological study. Lancet Infect Dis. 2017;17:1033–41. PubMed https://doi.org/10.1016/S1473-3099(17)30324-9

2. Lecorche E, Pean de Ponfilly G, Mougari F, Benmansour H, Poisnel E, Janvier F, et al. Disseminated *Mycobacterium chimaera* following open-heart surgery, the heater-cooler unit worldwide outbreak: case report and minireview. Front Med (Lausanne). 2020;7:243. PubMed https://doi.org/10.3389/fmed.2020.00243

3. Singh AK, Karaulia P, Chopra S, Dasgupta A. Draft genome sequence of *Mycobacterium fortuitum* isolated from murine brain. Genome Announc. 2016;4:e00191–16. PubMed https://doi.org/10.1128/genomeA.00191-16

4. Jaén-Luchoro D, Salvà-Serra F, Aliaga-Lozano F, Seguí C, Busquets A, Ramírez A, et al. Complete genome sequence of *Mycobacterium chelonae* type strain CCUG 47445, a rapidly growing species of nontuberculous mycobacteria. Genome Announc. 2016;4:e00550–16. PubMed https://doi.org/10.1128/genomeA.00550-16

5. Stinear TP, Seemann T, Harrison PF, Jenkin GA, Davies JK, Johnson PD, et al. Insights from the complete genome sequence of *Mycobacterium marinum* on the evolution of *Mycobacterium tuberculosis.* Genome Res. 2008;18:729–41. PubMed https://doi.org/10.1101/gr.075069.107

6. Peng F, Cheng X, Wang H, Song S, Chen T, Li X, et al. Structure-based reconstruction of a *Mycobacterium* hypothetical protein into an active Δ^5-3-ketosteroid isomerase. Biochim Biophys Acta Proteins Proteomics. 2019;1867:821–30. PubMed https://doi.org/10.1016/j.bbapap.2019.06.008
